# From pixels to patient care: deep learning-enabled pathomics signature offers precise outcome predictions for immunotherapy in esophageal squamous cell cancer

**DOI:** 10.1186/s12967-024-04997-z

**Published:** 2024-02-22

**Authors:** Butuo Li, Wenru Qin, Linlin Yang, Haoqian Li, Chao Jiang, Yueyuan Yao, Shuping Cheng, Bing Zou, Bingjie Fan, Taotao Dong, Linlin Wang

**Affiliations:** 1 Department of Radiation Oncology, Shandong Cancer Hospital and Institute, Shandong First Medical University and Shandong Academy of Medical Sciences, 440 Jiyan Road, Jinan, 250117 Shandong China; 2 Department of Otorhinolaryngology Head and Neck Surgery, Shandong Provincial Hospital Affiliated to Shandong First Medical University, Shandong Provincial Hospital, Cheeloo College of Medicine, Shandong University, Jinan, 250021 Shandong China; 3 Department of Obstetrics and Gynecology, Qilu Hospital of Shandong University, 107 West Wenhua Road, Jinan, 250063 Shandong China

**Keywords:** Pathomics, Esophageal cancer, Immunotherapy, Deep learning, Survival

## Abstract

**Background:**

Immunotherapy has significantly improved survival of esophageal squamous cell cancer (ESCC) patients, however the clinical benefit was limited to only a small portion of patients. This study aimed to perform a deep learning signature based on H&E-stained pathological specimens to accurately predict the clinical benefit of PD-1 inhibitors in ESCC patients.

**Methods:**

ESCC patients receiving PD-1 inhibitors from Shandong Cancer Hospital were included. WSI images of H&E-stained histological specimens of included patients were collected, and randomly divided into training (70%) and validation (30%) sets. The labels of images were defined by the progression-free survival (PFS) with the interval of 4 months. The pretrained ViT model was used for patch-level model training, and all patches were projected into probabilities after linear classifier. Then the most predictive patches were passed to RNN for final patient-level prediction to construct ESCC-pathomics signature (ESCC-PS). Accuracy rate and survival analysis were performed to evaluate the performance of ViT-RNN survival model in validation cohort.

**Results:**

163 ESCC patients receiving PD-1 inhibitors were included for model training. There were 486,188 patches of 1024*1024 pixels from 324 WSI images of H&E-stained histological specimens after image pre-processing. There were 120 patients with 227 images in training cohort and 43 patients with 97 images in validation cohort, with balanced baseline characteristics between two groups. The ESCC-PS achieved an accuracy of 84.5% in the validation cohort, and could distinguish patients into three risk groups with the median PFS of 2.6, 4.5 and 12.9 months (P < 0.001). The multivariate cox analysis revealed ESCC-PS could act as an independent predictor of survival from PD-1 inhibitors (P < 0.001). A combined signature incorporating ESCC-PS and expression of PD-L1 shows significantly improved accuracy in outcome prediction of PD-1 inhibitors compared to ESCC-PS and PD-L1 anlone, with the area under curve value of 0.904, 0.924, 0.610 for 6-month PFS and C-index of 0.814, 0.806, 0.601, respectively.

**Conclusions:**

The outcome supervised pathomics signature based on deep learning has the potential to enable superior prognostic stratification of ESCC patients receiving PD-1 inhibitors, which convert the images pixels to an effective and labour-saving tool to optimize clinical management of ESCC patients.

**Supplementary Information:**

The online version contains supplementary material available at 10.1186/s12967-024-04997-z.

## Introduction

Esophageal cancer ranked seventh in incidence and sixth in mortality worldwide [1]. Esophageal squamous cell carcinoma (ESCC) is the predominant type, accounting for 90% of all cases in East Asia and Africa[2]. Despite the gradual improvement in survival, the 5-year relative survival rate of ESCC patients remains less than 20% [3].

Immunotherapy have revolutionized the treatment schemes across ESCC patients [4]. The phase III KEYNOTE-181 [4] and ATTRACTION-3 [5] trials have demonstrated better efficacy and tolerable adverse effect with immune checkpoint inhibitors (ICIs) in advanced ESCC patients compared with conventional chemotherapy. It should be noted that the clinical benefit of ICIs was limited to only a small portion of ESCC patients, and a subset of patients might experience rapid tumor progression after receiving ICIs [5]. This suggests the importance to identify biomarkers to predict which patients could benefit from ICIs.

Expression of PD-L1 is now currently considered as a predictive marker for ICIs [6]. Nonetheless, the predictive value of PD-L1 status in ESCC is still controversial [7]. Checkmate 648 indicated that patients with PD-L1 of 1% or higher was associated with a significant progression-free survival (PFS) benefit after receiving ICIs [8]. However, ESCORT-1st trial indicated no significant correlation between PD-L1 status and efficacy of camrelizumab in ESCC patients [9]. It follows that single biomarker could not adequate for the accurate prediction of outcomes of PD-1 inhibitors.

Pathology, which has traditionally been employed as the basis of diagnosis, is the cornerstone of modern medicine and cancer care. Moreover, the pathology of tumor could reflect the heterogeneous characteristics of the tumor microenvironment (TME), and has been found to have the ability to prognosis prediction [10]. The development of the digital slide scanners advanced whole slide images (WSIs) from pathological slides which are high-resolution panoramic images contains cell structure and stroma. Employing the rich information of WSIs, computational pathology provided insights into the TME and facilitate computer-assisted diagnostics to alleviated the labour-intensive efforts of pathologists [11].

In the new era of artificial intelligence oncology, deep learning-based pathology can not only assist in image classification tasks, but also prognosis prediction by extracting risk-related histopathological features to identify intricate patterns and biological characteristics [12]. Jiang et al. performed a GC-SVM classifier using immunomarkers in immunohistochemistry staining slices and demonstrated the ability to predict the adjuvant chemotherapy benefit of gastric cancer patients [13]. Besides, a convolutional neural network-based classifier based on H&E images was also demonstrated to perform well in prognosis prediction of stage III colon cancer patients [14]. Although these prognosis prediction studies achieved promising performance using pathological images, the important role of pathomics to predict the clinical benefit from immunotherapy was largely unknown. Thus, we aimed to perform a deep learning-based pathomics signature using ViT-RNN network to accurately predict the clinical benefit of immunotherapy in ESCC patients.

## Material and methods

### Patient cohorts and data resource

ESCC patients receiving PD-1 inhibitors between 1 January 2018 and 1 January 2023 at Shandong Cancer Hospital and institute were included in this study. The inclusion criteria were as follows: (1) pathologically and radiological diagnosed as esophageal squamous cancer patients; (2) receiving PD-1 inhibitors; (3) with access to survival follow-up data. Patients with another primary malignant neoplasms were excluded from further analysis. The baseline characteristics were collected, including age, gender, smoking history, drinking history, TNM stage, metastasis and radiotherapy. Besides, the H&E-stained histological specimens of included patients were collected. The WSI images of H&E-stained histological specimens from included patients were scanned using Pannoramic MIDI II, Pannoramic SCAN II scanner or Zeiss Axio Scan.Z1. All images were saved as TIF files in pathological dataset, and were randomly divided into training (70%) and validation (30%) sets. The detailed clinical characteristics and survival time of the cohort are also retrieved. Immunohistochemistry staining for PD-L1 was performed on FFPE tumour tissue using PD-L1(22C3) monoclonal antibodies. The expression of PD-L1 was measured by tumor proportion score (TPS), which is defined as the percentage of membrane-positive tumor cells in all tumor cells.

### Label generation

The primary endpoint of the study was PFS of included patients after receiving PD-1 inhibitors, which was defined as the time from the beginning of PD-1 inhibitors to the disease progression or death. And the secondary endpoint was overall survival (OS) of included patients after receiving PD-1 inhibitors, which was defined as the time from the beginning of PD-1 inhibitors to the death. The PFS time of the cohort was used as labels for further model training. In this study, the survival prediction from PD-1 inhibitors was formulated as a classification problem.

### WSI images pre-processing

After digitization, the WSI images were pre-processed for further patch-level and patient-level model training. We split the WSI into small patches of 1024 × 1024 pixels at 20 × magnification. To eliminate unnecessary white background in the further process, we selected patches with variable of pixels more than 500. An additional challenge of these images was the stain color distribution differed from WSIs due to the complex staining process, which we chose to address slide level color normalization using Macenko method.

### Patch-level model training

Firstly, the ViT_base_patch_16_384 architecture which has been pretrained in ImageNet dataset was used for patch-level model training. The input data were the patch images obtained from splitting the WSI, and the labels of patches were the same as the WSI image of respective patient. In order to reduce the influence of noise and prevent overfitting, symmetric cross-entropy was applied to calculate the loss. Adam optimizer was used as the optimizer algorithm with an initial learn rate of 0.0001, weight decay of 0.0001 and a 50-image batch size. The patch level model was trained for 50 epochs.

### Prediction probability distribution of patches in patient level

To obtain the patient-level probability distribution of patches, the softmax output vectors were used to train a linear classifier. All patches from the WSI image of a single patient were summed together, and projected into probabilities using the linear classifier. The patches were ranked by their prediction probabilities, and the top 100 patches with highest probabilities were selected and assigned to the patient. Then a feature extractor would be trained for patches selection.

### Training and validation of patient-level pathomics signature

**Fig. 1:**
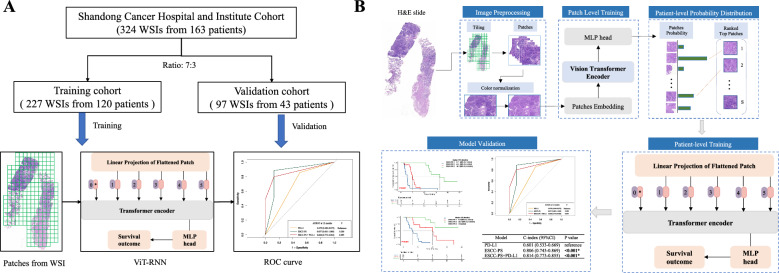
The overall workflow of ESCC-PS construction. **A** The process of sample splitting. **B** Schematic illustration of histopathology image processing and ESCC-PS construction based on ViT-RNN. *ESCC-PS* esophageal squamous cell cancer-pathomics signature, *ViT-RNN* Vision Transformer-Recursive Neural Network

The 40 most suspicious patches of each WSI image are sequentially passed to the RNN for features integration and final patient-level prediction. The cross-entropy was used to calculate the loss of RNN model, and Adam optimizer was used as the optimizer algorithm with a batch size of 2. The ESCC-pathomics signature (ESCC-PS) was constructed based on the patient-level ViT-RNN. All patients were assigned to three groups according to the ESCC-PS, and the accuracy rate were calculated to evaluate the performance in validation cohort. Univariate and multivariate survival analysis was performed to confirm the predictive effect of ESCC-PS in validation cohort. The whole process of this study was shown in Fig. 1.

### Incremental value of ESCC-PS for expression of PD-L1

The expression of PD-L1 was evaluated using immunohistochemical stains. And the cut-off values of PD-L1 was determined by receiver operating characteristic (ROC) curve in training cohort, in order to divide patients into high and low expression group. The predictive effect of PD-L1 was evaluated by cox regression analysis. ESCC-PS incorporating expression of PD-L1 for the outcome prediction of PD-1 inhibitors were applied to determine the incremental value of ESCC-PS. Patients in validation cohort were divided into low-risk, medium-risk and high-risk group based on the incorporation of ESCC-PS and PD-L1. The performance of PD-L1, ESCC-PS and incorporation signature were assessed and compared by C-index in validation cohort.

### Statistical analysis

The construction of ESCC-PS was performed using Python 3.6. And SPSS 26 and R 4.3.1 were used to conduct the data analysis and visualization. Kaplan–Meier survival was carried out to verify the clinical significance of ESCC-PS. Multivariable analysis was conducted using Cox proportional hazards modeling to validate the predictive value of ESCC-PS. Interactions between ESCC-PS and patient characteristics were detected by χ2 test. ROC curve with area under the curve (AUC) was performed to compare the performance between PD-L1, ESCC-PS and incorporation signature. All tests were 2-sided, and P < 0.05 was considered to indicate statistical significance.

## Results

### Clinical characteristics of patients with ESCC receiving immunotherapy

There were 163 ESCC patients receiving PD-1 inhibitors from Shandong Cancer Hospital with baseline data and known outcomes included in analysis. 324 WSI images of H&E-stained slides were retrieved from these patients, and were randomly divided into training cohort and validation cohort with the proportion of 7:3. The patches from a single patient were divided into the same cohort. There were 120 patients with 227 images in training cohort and 43 patients with 97 images in validation cohort. The clinical characteristics of patients in this cohort were illustrated in Table 1, and no significant difference was observed between training and validation cohort. After the median follow-up time of 14.2 months, the median PFS was 7.9 months for the whole patients. The optimal cut-off of PD-L1 expression was set as 57.5% with the AUC of 0.632 in training cohort.

**Table 1:** Baseline characteristics of patients in training cohort and validation cohort

Patient characteristics	Training Cohort (N = 120)	Validation Cohort (N = 43)	P value
Age (Median)	61.21	60.35	0.582
Gender			
Male	108 (90.0%)	34 (79.1%)	0.066
Female	12 (10.0%)	9 (20.9%)	
Smoking history			
No	57 (47.5%)	25 (58.1%)	0.231
Yes	63 (52.5%)	18 (41.9%)	
Drinking history			
No	72 (60.0%)	23 (53.5%)	0.457
Yes	48 (40.0%)	20 (46.5%)	
T stage			
T1-T2	29 (24.2%)	5 (11.6%)	0.083
T3-T4	91 (75.8%)	38 (88.4%)	
N stage			
N0-N1	59 (49.2%)	17 (39.5%)	0.277
N2-N3	61 (50.8%)	26 (60.5%)	
Stage			
III	31 (25.8%)	13 (30.2%)	0.577
IV	89 (74.2%)	30 (69.8%)	
Lung metastasis			
No	101 (84.2%)	34 (79.1%)	0.447
Yes	19 (15.8%)	9 (20.9%)	
Brain metastasis			
No	118 (98.3%)	43 (100.0%)	> 0.999
Yes	2 (1.7%)	0 (0%)	
Bone metastasis			
No	110 (91.7%)	41 (95.3%)	0.734
Yes	10 (8.3%)	2 (4.7%)	
Liver metastasis			
No	104 (86.7%)	38 (88.4%)	0.775
Yes	16 (13.3%)	5 (11.6%)	
Radiotherapy			
No	32 (26.7%)	12 (27.9%)	0.875
Yes	88 (73.3%)	31 (72.1%)	
Chemotherapy			
No	23 (19.2%)	12 (27.9%)	0.231
Yes	97 (80.8%)	31 (72.1%)	
PD-L1			
< 57.5%	26 (63.4%)	34 (79.1%)	0.112
≥ 57.5%	15 (36.6%)	9 (20.9%)	
ESCC-PS			
ESCC-PS 1	32 (26.7%)	19 (44.2%)	0.071
ESCC-PS 2	57 (47.5%)	13 (30.2%)	
ESCC-PS 3	31 (25.8%)	11 (25.6%)	

### The training of ViT-RNN survival model and construction of ESCC-PS

There were 486,188 patches with 1024 × 1024 pixels after image pre-processing from 324 WSI images. Then subsampling was used to resize these patches into 384*384 pixels to adapt to pretrained ViT_base_patch_16_384. All patches were used as input to ViT for patch-level model training, then were projected into group probabilities. The 40 most predictive patches are sequentially passed to the ViT-RNN for final patient-level prediction and construction of ESCC-PS. Patients in validation cohort were projected into three groups based on ESCC-PS, and 19 patients were projected in ESCC-PS 1 group, 13 patients in ESCC-PS 2 group and 11 patients in ESCC-PS 3 group. As shown in Table 2, no potential interactions were found between ESCC-PS and patient characteristics in validation cohort.

**Table 2:** Correlation analysis between ESCC-PS and patient characteristics

Patient characteristics	ESCC-PS 1 (N = 19)	ESCC-PS 2 (N = 13)	ESCC-PS 3 (N = 11)	P value
Age				
≤ 60	10 (52.6%)	8 (61.5%)	6 (54.5%)	0.879
> 60	9 (47.4%)	5 (38.5%)	5 (45.5%)	
Gender				
Male	15 (78.9%)	11 (84.6%)	8 (72.7%)	0.892
Female	4 (21.1%)	2 (15.4%)	3 (27.3%)	
Smoking history				
No	12 (63.2%)	7 (53.8%)	6 (54.5%)	0.838
Yes	7 (36.8%)	6 (46.2%)	5 (45.5%)	
Drinking history				
No	9 (47.4%)	8 (61.5%)	6 (54.5%)	0.730
Yes	10 (52.6%)	5 (38.5%)	5 (45.5%)	
T stage				
T1-T2	1 (5.3%)	3 (23.1%)	1 (9.1%)	0.413
T3-T4	18 (94.7%)	10 (76.9%)	10 (90.9%)	
N stage				
N0-N1	5 (26.3%)	8 (61.5%)	4 (36.4%)	0.131
N2-N3	14 (73.7%)	5 (38.5%)	7 (63.6%)	
Stage				
III	6 (31.6%)	5 (38.5%)	2 (18.2%)	0.581
IV	13 (68.4%)	8 (61.5%)	9 (81.8%)	
Lung metastasis				
No	14 (73.7%)	11 (84.6%)	9 (81.8%)	0.892
Yes	5 (26.3%)	2 (15.4%)	2 (18.2%)	
Bone metastasis				
No	18 (94.7%)	13 (100.0%)	10 (90.9%)	0.726
Yes	1 (5.3%)	0 (0.0%)	1 (9.1%)	
Liver metastasis				
No	16 (84.2%)	13 (100.0%)	9 (81.8%)	0.351
Yes	3 (15.8%)	0 (0.0%)	2 (18.2%)	
Radiotherapy				
No	8 (42.1%)	2 (15.4%)	2 (18.2%)	0.217
Yes	11 (57.9%)	11 (84.6%)	9 (81.8%)	
Chemotherapy				
No	5 (26.3%)	4 (30.8%)	3 (27.3%)	0.961
Yes	14 (73.7%)	9 (69.2%)	8 (72.7%)	
PD-L1				
< 57.5%	17 (89.5%)	10 (76.9%)	7 (63.6%)	0.218
≥ 57.5%	2 (10.5%)	3 (23.1%)	4 (36.4%)	

### Test performance of ESCC-PS for outcome prediction of PD-1 inhibitors in validation cohort

**Fig. 2:**
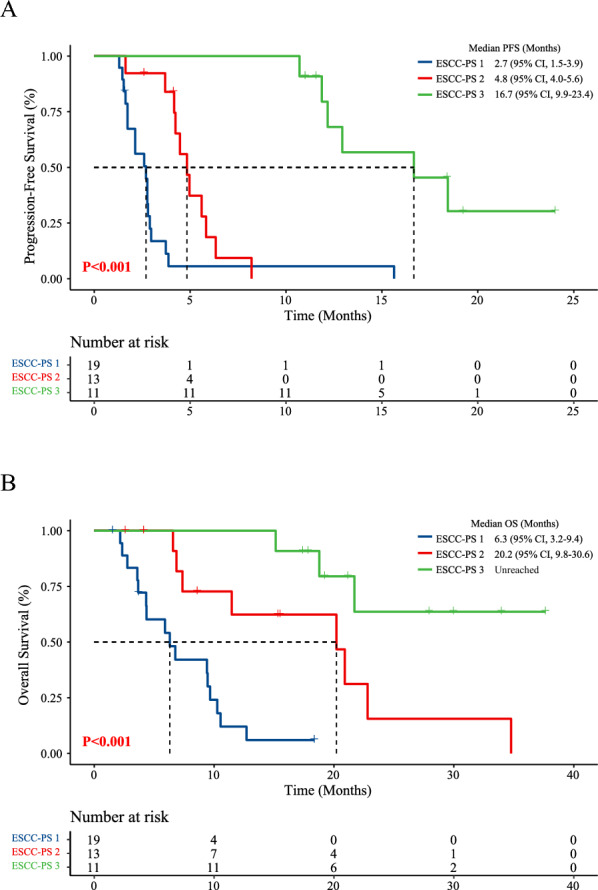
Kaplan–Meier survival curves according to the ESCC-PS in validation cohort.** A** Kaplan–Meier survival curves of PFS (2.7 vs 4.8 vs 16.7 months, P < 0.001) according to the ESCC-PS. B. Kaplan–Meier survival curves of OS (6.3 vs 20.2 months vs Unreached, P < 0.001) according to the ESCC-PS. *PFS* progression-free survival, *OS* overall survival, *ESCC-PS* esophageal squamous cell cancer-pathomics signature

The ViT-RNN based ESCC-PS achieved the accuracy of 92% in training cohort, and 84.5% in validation cohort. Four iterations of random partition of all patients were performed to investigate the stability of the model, and the accuracies of the model ranged from 82.7% to 95.1% in the validation cohort, indicated the stability of the model. The Kaplan Meier survival curves indicated the significant difference on the PFS (P < 0.001) with the median PFS of 2.7, 4.8 and 16.7 months respectively between ESCC-PS 1, ESCC-PS 2, and ESCC-PS 3 group (Fig. 2A). The superiority in OS (P < 0.001) was found for patients in ESCC-PS 3 group (Unreached) compared to ESCC-PS 1 (6.3 month) and 2 group (20.2 month) (Fig. 2B). And the predictive effect of ESCC-PS (P < 0.001) was also shown according to the univariate cox analysis, highlighting the predictive value of ViT-RNN based ESCC-PS. As shown in Table 3, the expression of PD-L1 was also associated with the PFS of ESCC patients receiving immunotherapy (HR = 0.486, P = 0.092). Multivariate cox analysis indicated both PD-L1 (HR = 0.231, P = 0.009) and ESCC-PS (P < 0.001) remained a significant prognostic indicator indicating the independent prognostic factors for ESCC patients receiving immunotherapy. The superior performance of ESCC-PS for the prediction of PFS (P < 0.001) and OS (P = 0.005) was also shown in the training cohort based on multivariate cox regression analysis (Additional file 1: Tables S1 and S2).

**Table 3:** Univariate and multivariate cox regression analysis of ESCC-PS and clinicopathological characteristics for progression-free survival in validation cohort

Patient characteristics	Univariate Cox analysis		Multivariate Cox analysis	
	HR (95%CI)	P value	HR (95%CI)	P value
Age				
≤ 60	1	0.977		
> 60	0.990 (0.507–1.935)			
Gender				
Male	1	0.686		
Female	0.847 (0.378–1.896)			
Smoking history				
No	1	0.955		
Yes	1.021 (0.501–2.081)			
Drinking history				
No	1	0.497		
Yes	1.277 (0.631–2.584)			
T stage				
T1-T2	1	0.895		
T3-T4	1.067 (0.411–2.766)			
N stage				
N0-N1	1	0.984		
N2-N3	0.993 (0.504–1.959)			
Stage				
III	1	0.603		
IV	1.219 (0.579–2.566)			
Lung metastasis				
No	1	0.427		
Yes	1.412 (0.603–3.311)			
Bone metastasis				
No	1	0.996		
Yes	1.003 (0.235–4.288)			
Liver metastasis				
No	1	0.915		
Yes	1.059 (0.371–3.025)			
Radiotherapy				
No	1			
Yes	0.543 (0.249–1.185)	0.125		
Chemotherapy				
No	1	0.771		
Yes	1.121 (0.519–2.420)			
PD-L1				
< 57.5%	1	0.092*	1	0.009*
≥ 57.5%	0.486 (0.210–1.124)		0.231 (0.077–0.696)	
ESCC-PS				
ESCC-PS 1	1	< 0.001*	1	< 0.001*
ESCC-PS 2	0.393 (0.172–0.897)		0.275 (0.108–0.700)	
ESCC-PS 3	0.055 (0.018–0.173)		0.029 (0.008–0.111)	

*Statistcal significance

### Incremental value of the ESCC-PS added to the expression of PD-L1 for outcome prediction

**Fig. 3:**
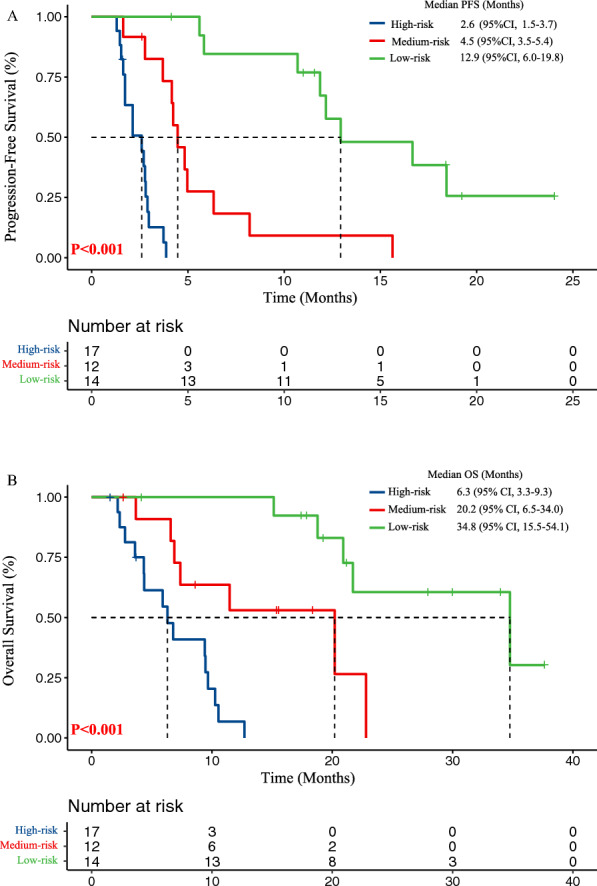
Kaplan–Meier survival curves according to the incorporation of ESCC-PS and PD-L1 in validation cohort. **A** Kaplan–Meier survival curves of PFS (2.6 vs 4.5 vs 12.9 months, P < 0.001) according to the incorporation of ESCC-PS and PD-L1. B. Kaplan–Meier survival curves of OS (6.3 vs 20.2 vs 34.8 months, P < 0.001) according to the incorporation of ESCC-PS and PD-L1. *PFS* progression-free survival, *OS* overall survival, *ESCC-PS* esophageal squamous cell cancer-pathomics signature

The incorporation of ESCC-PS and expression of PD-L1 was performed based on the multivariate cox regression analyses to elucidate the incremental value of the ESCC-PS added to the expression of PD-L1 for predicting the outcome of PD-1 inhibitors. As shown in Fig. 3, survival analysis indicated the incorporation signature could significantly distinguish patients into high-risk, medium-risk and low-risk, with the PFS of 2.6, 4.5, 12.9 months, and OS of 6.3, 20.2, 34.8 months, respectively (Table 4)

**Table 4:** Univariate and multivariate cox regression analysis of ESCC-PS and clinicopathological characteristics for overall survival in validation cohort

Patient characteristics	Univariate Cox analysis		Multivariate Cox analysis	
	HR (95%CI)	P value	HR (95% CI)	P value
Age				
≤ 60	1	0.495		
> 60	1.309 (0.604–2.838)			
Gender				
Male	1	0.906		
Female	0.947 (0.379–2.365)			
Smoking history				
No	1	0.660		
Yes	1.192 (0.546–2.602)			
Drinking history				
No	1	0.214		
Yes	1.641 (0.751–3.583)			
T stage				
T1-T2	1	0.644		
T3-T4	1.328 (0.398–4.434)			
N stage				
N0-N1	1	0.482		
N2-N3	0.755 (0.345–1.653)			
Stage				
III	1	0.673		
IV	1.205 (0.507–2.862)			
Lung metastasis				
No	1	0.172		
Yes	1.834 (0.768–4.382)			
Bone metastasis				
No	1	0.494		
Yes	1.666 (0.386–7.198)			
Liver metastasis				
No	1	0.460		
Yes	1.497 (0.513–4.362)			
Radiotherapy				
No	1	0.025*		
Yes	0.386 (0.168–0.888)			
Chemotherapy				
No	1	0.981		
Yes	1.011 (0.423–2.16)			
PD-L1				
< 57.5%	1	0.037*	1	0.041*
≥ 57.5%	0.214 (0. 050–0.909)		0.213 (0.048–0.942)	
ESCC-PS				
ESCC-PS 1	1	< 0.001*	1	< 0.001*
ESCC-PS 2	0.167 (0.056–0.496)		0.168 (0.055–0.516)	
ESCC-PS 3	0.040 (0.009–0.171)		0.034 (0.007–0.160)	

*Statistcal significance

**Fig. 4:**
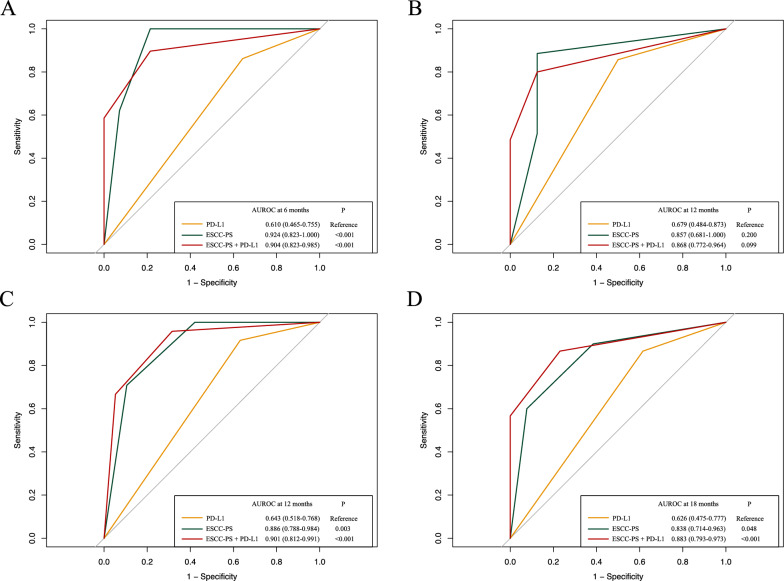
The comparison of ROC curves for survival of PD-L1, ESCC-PS and ESCC-PS + PD-L1 in validation cohort. **A** The comparison of ROC curves for evaluating 6-month PFS (AUROC: 0.610, 0.924 and 0.904). **B** The comparison of ROC curves for evaluating 12-month PFS (AUROC: 0.679, 0.857 and 0.868). **C** The comparison of ROC curves for evaluating 12-month OS (AUROC: 0.643, 0.886 and 0.901). D. The comparison of ROC curves for evaluating 18-month OS (AUROC: 0.626, 0.838 and 0.883). *ROC* receiver operating characteristic, *AUROC* area under ROC curve, *PFS* progression-free survival, *OS* overall survival, *ESCC-PS* esophageal squamous cell cancer-pathomics signature

The C-index of ESCC-PS for PFS after receiving PD-1 inhibitors was 0.806, which was significantly higher than that of the expression of PD-L1 (0.601, P < 0.001). As shown in Table 5, the significantly incremental C-index was observed for incorporation of ESCC-PS and PD-L1 compared with PD-L1 alone (0.814 vs 0.601, P < 0.001). The comparison of ROC for prediction of PFS and OS between PD-L1, ESCC-PS and ESCC-PS + PD-L1 was shown in Fig. 4. The AUC of ESCC-PS + PD-L1 for 6 month- (0.904 vs 0.610, P < 0.001) and 12 month- PFS (0.868 vs 0.679, P = 0.099) prediction was higher than PD-L1. Similarly, ESCC-PS + PD-L1 also exhibited higher AUC for prediction of 12 month- (0.901 vs 0.643, P < 0.001) and 18 month- OS (0.883 vs 0.626, P < 0.001).

**Table 5:** Comparison of C-index for progression-free survival in validation cohort

Model	C-index (95%CI)	P value
PD-L1	0.601 (0.533–0.669)	Reference
ESCC-PS	0.806 (0.743–0.869)	< 0.001*
ESCC-PS + PD-L1	0.814 (0.773–0.855)	< 0.001*

*Statistcal significance

## Discussion

Herein, we constructed a computational pathomics signature named ESCC-PS using H&E stained WSI images based on outcome supervised ViT-RNN to predict the survival of ESCC patients receiving PD-1 inhibitors. Validation experiments confirmed the excellent performance and independent predictive effect of this ESCC-PS, and the incremental value for the expression of PD-L1 for outcome prediction of PD-1 inhibitors.

The microscopic study including H&E-stained histopathological images was the cornerstone for cancer diagnosis and prognosis. However, the histological identificantion by pathologists faces challenges including the heterogeneity of tumor. Tumor with the same histology can develop in different prognosis, and tumor with different histology can develop in the same process. Some intrinsic pathological features which cannot be recognized by human eyes might have a greater impact on the development and prognosis of tumors. Besides, the extremely large spatial size of WSIs makes it difficult to extract hand-crafted features.

Deep learning, the state-of-the-art technique in computer vision, have presented the potential to automatically identify and analyze high throughout features in WSI, which were not limited to hand-crafted features from existing knowledge. The deep learning pathomics can be used for objective diagnosis, phenotype recognition and prognostic prediction, and realize the pathological pixel to patient care [15–17]. Shi etc. has established an interpretable pathomics model using Resnet-18 and quantified as “tumor risk score (TRS)” to predict the clinical outcomes of hepatocellular carcinoma patients [18]. Qaiser T, et al. constructed a weakly supervised survival convolutional neural network (WSS-CNN) approach equipped with a visual attention mechanism based on WSI images of H&E-stained specimens, and demonstrated its outperformed performance for outcome prediction of lung cancer patients with the C-index of 0.6863 [19].

In this study, the ESCC-PS based on WSI images of H&E-stained specimens were constructed for outcome prediction of PD-1 inhibitors, and the deep neural network for model training is vision transformer embedded self-attention mechanism, with image data as the input rather than manual feature extraction. Previous study has demonstrated the superior performance of ViT pretrained on Image-Net dataset, which was used in this study [20], compared to CNN. The output of survival prediction model was risk stratification by splitting the survival time into three discrete groups. The patches from WSI images including positional embedding were as the input of transformer encoder [21], which incorporated more spatial information at lower layers. And the self-attentional mechanism allows ViT to capture the global features of the image rather than the dependencies between adjacent elements. This pathomics signature using ViT-RNN prognostic model is expected the identify ESCC patients who might benefit from PD-1 inhibitors, and assist in the development of individual treatment strategies.

H&E-stained specimens were also found to characteristic TME to some extent based on deep learning, which could dig out more information for prognosis prediction [22, 23]. Jiao etc. has performed the CNN to recognize the stroma, tumor, necrosis, and lymphocyte components of TME from colon cancer H&E-stained specimens, and survival analysis also indicated the prognostic value of them in colorectal cancer [23]. The WSI of H&E-stained specimens contains the delicate details of the tissue. Deeep learning based pathomics could be a useful tool for the data mining including the features of cells, intercellular junction and others, which are more suitable for risk stratification and prognosis prediction and might be the reason for the outcome prediction of ESCC-PS. However, the interpretability and the predictive biological features for ESCC-PS model need further investigated in future studies.

Despite limited performance, expression of PD-L1 remains the cornerstone for predicting the outcome of ESCC patients receiving PD-1 inhibitors [24], which was also demonstrated in this study. Besides, we discovered the superiority performance of ESCC-PS compared to the expression of PD-L1. As the ESCC-PS was derived from the H&E-stained sections, which was routinely performed in the clinic, ESCC-PS might be conveniently applied without additional financial burden in clinical practice. In addition, significantly improved predictive performance was detected when the ESCC-PS was added to the expression of PD-L1, with the improvement in C-index ranged from 0.659 to 0.819. The result indicated the additional biological information from the ESCC-PS, and might favour the personalized application of PD-1 inhibitors.

There were still some limitations in this study. Firstly, further validation of this pathomics prognostic model was needed in external cohort. Besides, the black box feature of deep learning makes it difficult to explore the biological mechanism of ViT-RNN model for outcome prediction. The feasible way to explore the underlying mechanism of ESCC-PS with PD-1 inhibitors benefits needs further investigation. In addition, multi-omics-based outcome prediction might achieve better performance including but not limited to pathomics, radiomics and genomics.

In conclusion, we developed and verified a pathomics model named ESCC-PS based on ViT -RNN framework from WSIs. The ESCC-PS could act as an excellent predictor, and played complementary role of PD-L1 for outcome prediction of PD-1 inhibitors, which could aid the clinical decision making.

### Supplementary Information

**Additional file 1: Table S1.** Univariate and multivariate cox regression analysis of ESCC-PS and clinicopathological characteristics for progression-free survival in training cohort. **Table S2.** Univariate and multivariate cox regression analysis of ESCC-PS and clinicopathological characteristics for overall survival in training cohort.

## Data Availability

The data that support the findings of this study are available from the corresponding author upon reasonable request.

## Abbreviations

ESCC: Esophageal squamous cell cancer
ESCC-PS: Esophageal squamous cell cancer-pathomics signature
TME: Tumor microenvironment
ViT-RNN: Vision Transformer-Recursive Neural Network
PFS: Progression-free survival
OS: Overall survival
ROC: Receiver operating characteristic
AUROC: Area under ROC curve
ICIs: Immune checkpoint inhibitors
WSIs: Whole slide images
TRS: Tumor risk score
WSS-CNN: Weakly supervised survival convolutional neural network
