# Supplementary material for: From pixels to patient care: deep learning-enabled pathomics signature offers precise outcome predictions for immunotherapy in esophageal squamous cell cancer
Source: J Transl Med. 2024 Feb 22;22:195. doi: 10.1186/s12967-024-04997-z (PMC10885627; doi:10.1186/s12967-024-04997-z)
Supplement: Supplementary file 1 — Additional file 1: Table S1. Univariate and multivariate cox regression analysis of ESCC-PS and clinicopathological characteristics for progression-free survival in training cohort. Table S2. Univariate and multivariate cox regression analysis of ESCC-PS and clinicopathological characteristics for overall survival in training cohort. [file 12967_2024_4997_MOESM1_ESM.docx]

**Table 1 Univariate and multivariate cox regression analysis of ESCC-PS and clinicopathological characteristics for progression-free survival in training cohort**

| Patient characteristics | Univariate Cox analysis | | Multivariate Cox analysis | |
| --- | --- | --- | --- | --- |
|  | HR (95%CI) | P value | HR (95%CI) | P value |
| Age |  |  |  |  |
| ≤60 | 1 | 0.290 |  |  |
| >60 | 0.802 (0.532-1.207) |  |  |  |
| Gender |  |  |  |  |
| Male | 1 | 0.102 |  |  |
| Female | 0.498 (0.216-1.148) |  |  |  |
| Smoking history |  |  |  |  |
| No | 1 | 0.235 |  |  |
| Yes | 1.286 (0.849-1.946) |  |  |  |
| Drinking history |  |  |  |  |
| No | 1 | 0.672 |  |  |
| Yes | 1.094 (0.722-1.659) |  |  |  |
| T stage |  |  |  |  |
| T1-T2 | 1 | 0.656 |  |  |
| T3-T4 | 1.120 (0.681-1.840) |  |  |  |
| N stage |  |  |  |  |
| N0-N1 | 1 | **0.007*** | 1 | **0.010*** |
| N2-N3 | 1.767 (1.166-2.678) |  | 1.768 (1.147-2.723) |  |
| Stage |  |  |  |  |
| III | 1 | **0.014*** | 1 | **0.016*** |
| IV | 1.863 (1.132-3.065) |  | 1.912 (1.129-3.238) |  |
| Lung metastasis |  |  |  |  |
| No | 1 | 0.620 |  |  |
| Yes | 1.155 (0.653-2.044) |  |  |  |
| Bone metastasis |  |  |  |  |
| No | 1 | 0.123 |  |  |
| Yes | 1.724 (0.864-3.441) |  |  |  |
| Liver metastasis |  |  |  |  |
| No | 1 | 0.058 |  |  |
| Yes | 1.715 (0.982-2.995) |  |  |  |
| Radiotherapy |  |  |  |  |
| No | 1 | 0.390 |  |  |
| Yes | 0.817 (0.515-1.296) |  |  |  |
| PD-L1 |  |  |  |  |
| <57.5% | 1 | 0.410 |  |  |
| ≥57.5% | 0.744 (0.369-1.502) |  |  |  |
| Chemotherapy |  |  |  |  |
| No | 1 | 0.776 |  |  |
| Yes | 0.930 (0.565-1.532) |  |  |  |
| ESCC-PS |  |  |  |  |
| ESCC-PS 1 | 1 | ＜**0.001*** | 1 | ＜**0.001*** |
| ESCC-PS 2 | 0.476 (0.295-0.768) |  | 0.369 (0.222-0.612) |  |
| ESCC-PS 3 | 0.215 (0.119-0.388) |  | 0.171 (0.093-0.317) |  |

**Table 2 Univariate and multivariate cox regression analysis of ESCC-PS and clinicopathological characteristics for overall survival in training cohort**

| Patient characteristics | Univariate Cox analysis | | Multivariate Cox analysis | |
| --- | --- | --- | --- | --- |
|  | HR (95%CI) | P value | HR (95%CI) | P value |
| Age |  |  |  |  |
| ≤60 | 1 | 0.765 |  |  |
| >60 | 1.071 (0.683-1.679) |  |  |  |
| Gender |  |  |  |  |
| Male | 1 | 0.069 |  |  |
| Female | 0.392 (0.143-1.074) |  |  |  |
| Smoking history |  |  |  |  |
| No | 1 | **0.043*** | 1 | **0.038*** |
| Yes | 1.607 (1.016-2.543) |  | 1.628 (1.027-2.581) |  |
| Drinking history |  |  |  |  |
| No | 1 | 0.224 |  |  |
| Yes | 1.324 (0.843-2.079) |  |  |  |
| T stage |  |  |  |  |
| T1-T2 | 1 | 0.300 |  |  |
| T3-T4 | 1.338 (0.771-2.323) |  |  |  |
| N stage |  |  |  |  |
| N0-N1 | 1 | **0.047*** |  |  |
| N2-N3 | 1.586 (1.005-2.501) |  |  |  |
| Stage |  |  |  |  |
| III | 1 | **0.028*** | 1 | **0.025*** |
| IV | 1.866 (1.069-3.258) |  | 1.905 (1.083-3.348) |  |
| Lung metastasis |  |  |  |  |
| No | 1 | 0.807 |  |  |
| Yes | 0.924 (0.488-1.750) |  |  |  |
| Bone metastasis |  |  |  |  |
| No | 1 | 0.069 |  |  |
| Yes | 1.986 (0.949-4.158) |  |  |  |
| Liver metastasis |  |  |  |  |
| No | 1 | **0.029*** |  |  |
| Yes | 1.960 (1.070-3.591) |  |  |  |
| Radiotherapy |  |  |  |  |
| No | 1 | 0.390 |  |  |
| Yes | 0.817 (0.515-1.296) |  |  |  |
| PD-L1 |  |  |  |  |
| <57.5% | 1 | 0.997 |  |  |
| ≥57.5% | 0.999 (0.491-2.031) |  |  |  |
| Chemotherapy |  |  |  |  |
| No | 1 | 0.863 |  |  |
| Yes | 0.953 (0.548-1.654) |  |  |  |
| ESCC-PS |  |  |  |  |
| ESCC-PS 1 | 1 | **0.006*** | 1 | **0.005*** |
| ESCC-PS 2 | 0.710 (0.420-1.201) |  | 0.650 (0.383-1.103) |  |
| ESCC-PS 3 | 0.341 (0.176-0.660) |  | 0.332 (0.171-0.646) |  |
